# Laparotomy in women with severe acute maternal morbidity: secondary analysis of a nationwide cohort study

**DOI:** 10.1186/s12884-018-1688-2

**Published:** 2018-02-27

**Authors:** Tom Witteveen, Athanasios Kallianidis, Joost J. Zwart, Kitty W. Bloemenkamp, Jos van Roosmalen, Thomas van den Akker

**Affiliations:** 1 Department of Obstetrics, Leiden University Medical Center, building 1, room K-6-P-35, P.O. Box 9600, 2300 RC Leiden, The Netherlands; 2 Department of Obstetrics and Gynecology, Haga Teaching Hospital, Els Borst-Eilersplein 275, 2545 AA Den Haag, The Netherlands; 3 Department of Obstetrics and Gynecology, Deventer Ziekenhuis, Nico Bolkesteinlaan 75, 7416 SE Deventer, The Netherlands; 4 Department of Obstetrics, Wilhelmina Children’s Hospital Birth Centre, University Medical Centre Utrecht, Lundlaan 6, 3584 EA Utrecht, The Netherlands; 5 Athena Institute, Faculty of Science, VU University Amsterdam, De Boelelaan 1085, 1081 HV Amsterdam, The Netherlands

**Keywords:** Childbirth, Obstetric surgical procedures, Cesarean section, Laparotomy, Severe acute maternal morbidity, Maternal mortality, High-risk pregnancy, Obstetrics, Cohort studies

## Abstract

**Background:**

Although pregnancy-related laparotomy is a major intervention, literature is limited to small case-control or single center studies. We aimed to identify national incidence rates for postpartum laparotomy related to severe acute maternal morbidity (SAMM) in a high-income country and test the hypothesis that risk of postpartum laparotomy differs by mode of birth.

**Methods:**

In a population-based cohort study in all 98 hospitals with a maternity unit in the Netherlands, pregnant women with SAMM according to specified disease and management criteria were included from 01/08/2004 to 01/08/2006. We calculated the incidence of postpartum laparotomy after vaginal and cesarean births. Laparotomies were analyzed in relation to mode of birth using all births in the country as reference. Relative risks (RR) were calculated for laparotomy following emergency and planned cesarean section compared to vaginal birth, excluding laparotomies following births before 24 weeks’ gestation and hysterectomies performed during cesarean section.

**Results:**

The incidence of postpartum laparotomy in women with SAMM in the Netherlands was 6.0 per 10,000 births. Incidence was 30.1 and 1.8 per 10,000 following cesarean and vaginal birth respectively. Compared to vaginal birth, RR of laparotomy after cesarean birth was 16.7 (95% confidence interval [95% CI] 12.2-22.6). RR was 21.8 (95% CI 15.8-30.2) for emergency and 10.5 (95% CI 7.1-15.6) for planned cesarean section.

**Conclusions:**

Risk of laparotomy, although small, was considerably elevated in women who gave birth by cesarean section. This should be considered in counseling and clinical decision making.

## Background

According to the World Health Organization (WHO), laparotomy is a critical intervention required in the management of life-threatening and potentially life-threatening conditions [1]. In this study, laparotomy is defined as a surgical procedure involving an incision through the abdominal wall to gain access into the abdominal cavity other than cesarean section. Its use is indicative of severe maternal outcome and may be applied as a quality marker for obstetric care [1]. Although it is clear that laparotomy during pregnancy and after childbirth is a major intervention, literature is sparse and limited to case-control or single center studies with limited numbers of cases.

Previous studies only address ‘re-laparotomy’ after cesarean section. Reported incidence rates of ‘re-laparotomy’ are low, varying between 0.2 and 0.9% [2–11]. Although data on laparotomy after vaginal birth are not reported, it has been suggested that the incidence of laparotomy may be higher after cesarean section, since operative birth is associated with a higher risk of maternal morbidity and mortality [12, 13].

In this paper, we report national incidence rates of postpartum laparotomy, using a nationwide cohort of women with severe acute maternal morbidity (SAMM), and test the hypothesis that the risk of pregnancy-related laparotomy in the postpartum period differs by mode of birth.

## Methods

This study is part of a well-known two-year nationwide cohort study to assess SAMM during pregnancy, labour and puerperium in the Netherlands, called the ‘LEMMoN-study’ (Landelijke studie naar Ethnische determinanten van Maternale Morbiditeit in Nederland). Pregnant women sustaining SAMM were included from all 98 hospitals with a maternity unit, in the period 1st August 2004 until 1st August 2006. These were eight tertiary care hospitals, 35 non-academic teaching hospitals and 55 general hospitals. Detailed information regarding data collection was described previously [14].

Inclusion criteria for SAMM were categorized in five groups: admission into an intensive care unit, uterine rupture, eclampsia, major obstetric hemorrhage (defined as four or more units of pack red blood cells or hysterectomy or arterial embolization) and a miscellaneous group with SAMM in the opinion of the treating clinician, which could not be classified in any of the other four groups. Women could be included into more than one group, therefore: one woman could have more than one indication for laparotomy, and more than one comorbidity. For all calculations of risk and incidence, we used the number of women as the denominator. Laparotomy was not a specific inclusion criterion in the LEMMoN-study.

All women in the nationwide SAMM cohort who had a laparotomy after vaginal or cesarean birth were included in this specific study. Incidence of postpartum laparotomy and relative risks with regard to mode of birth were calculated. Only women with a birth after 24 weeks’ gestational age were included, and only those who had a laparotomy within six weeks after birth. Women who had hysterectomy or other surgery during cesarean birth were excluded.

The main outcome measure was relative risk (RR) related to cesarean birth (with vaginal birth as reference) and associated risk factors. The Dutch Perinatal Register was used as the source for background denominator data. Clinical characteristics and birth data were analyzed in search of predisposing factors. Maternal characteristics included age, body mass index, parity, gestational age, and previous cesarean section. Data concerning birth included: mode of birth, blood loss, number of units of blood transfused, indication for laparotomy, timing of laparotomy after birth (< 24 h, 2-7 days or > 7 days), number of laparotomies and duration of hospital admission. Indications for laparotomy were clustered into six groups: severe postpartum hemorrhage, intra-abdominal bleeding, (suspected) uterine rupture, sepsis, hematoma and miscellaneous (i.e. removal of purposely-left sterile gauze, bladder damage, rectovaginal fistula). Therapeutic interventions were clustered into: bleeding control, which was then subdivided by location (abdominal wall, intra-abdominal and uterine scar-related), compression sutures such as the B-lynch procedure, ligation of large vessels, hysterectomy, hematoma/abscess drainage, negative laparotomy (exploration without therapeutic intervention) and miscellaneous. More than one indication or intervention could be assigned.

RRs with 95% confidence intervals (CI) were calculated where appropriate. Differences in characteristics between modes of birth were tested with a chi-square test or Fisher’s exact test for categorical data and independent t-test or Mann-Whitney U test for numerical data where appropriate. Statistical analysis was performed using SPSS statistics, version 20.0 (SPSS, Chicago, IL).

## Results

During the two years, 355,841 births were registered in the Netherlands Perinatal Register: 302,689 (85.1%) vaginal births and 53,152 (14.9%) cesarean sections, of which 24,580 (46.2%) planned and 28,572 (53.8%) emergency sections. Among 2552 women with SAMM in the cohort, 325 laparotomies were reported in 276 women. This gives a total incidence of laparotomy in women with SAMM in the Netherlands of 7.8 per 10,000 births. Sixty-one women were excluded from analysis of risk as they did not fit the inclusion criteria: 37 had the (initial) laparotomy before birth, 15 had a cesarean section with additional procedures including 11 hysterectomies, 6 had delivered before 24 weeks’ gestational age and 3 were more than 42 days postpartum at the time of laparotomy.

The 215 remaining women were included for risk analysis, of whom 160 (74.4%) had laparotomies following cesarean section (10 out of these 160 were failed vacuum extractions) and 55 (25.6%) following vaginal birth (14 out of these 55 were instrumental births -all vacuum extractions, forceps are rarely used in the Netherlands).

One hundred and forty-five women (67.4%) were admitted into an intensive care unit. Comorbidity included major obstetric hemorrhage in 192 (89.3%), uterine rupture in 22 (10.2%), eclampsia in 8 (3.7%) and miscellaneous morbidity in six (2.8%) out of the 215 women. These ‘miscellaneous comorbidities’ were (A) postoperative adhesion ileus (twice), (B) large abdominal wall hematoma after cesarean section, (C) incarcerated hernia one day postpartum requiring ilio-caecal resection, (D) rectovaginal fistula nine days after anal sphincter rupture requiring colostomy, (E) a large wound defect with multiple abscesses.

One hundred thirty-eight women had more than one comorbidity (118 had two, 19 had three and one woman had four co-morbidities). The incidence of laparotomy after childbirth in women with SAMM in the Netherlands, who fitted our inclusion criteria for risk analysis in relation to mode of birth, was 6.0 per 10,000. Incidence was 30.1 per 10,000 cesarean births and 1.8 per 10,000 vaginal births (Table 1). This gives a RR of 16.7 (95% CI 12.2-22.6). The absolute risk of laparotomy was 39.5 per 10,000 births for emergency cesarean section and 19.1 per 10,000 for planned section. Compared to vaginal birth, RRs for emergency and planned cesarean section were 21.8 (95% CI 15.8-30.2) and 10.5 (95% CI 7.1-15.6) respectively (Table 1).

**Table 1:** Incidence of laparotomy after childbirth, related to mode of birth

		Births (*n*)	Laparotomy (*n*)	Incidence^*^	RR (95% CI)
Total		355,841	215	6.0	
CS		53,152	160	30.1	16.7 (12.2-22.6)
	Planned	24,580	47	19.1	10.5 (7.1-15.6)
	Emergency	28,572	113	39.5	21.8 (15.8-30.2)
VD		302,689	55	1.8	Reference

*RR* relative risk, *CI* confidence interval, *CS* cesarean section, *VD* vaginal birth

^*^per 10,000 births

Women who had laparotomy after cesarean section, were more often nulliparous, had pregnancies of lower gestational age and longer hospital admissions compared to those who gave birth vaginally (Table 2). Large proportions in both groups were found to have scarred uteri: 32.7% of women who delivered by cesarean section and 34.0% of women who delivered vaginally. Among women who had laparotomy after cesarean section the proportion of women with a scarred uterus secondary to previous cesarean section was larger in the planned cesarean section group (emergency 20.4%, planned 61.7%; *p* < 0.01). There were 103 women (48%) who needed to be transfused nine or more units of red blood cell concentrates: 30 following vaginal and 73 following cesarean birth.

**Table 2:** Maternal characteristics and birth information

	VD *N* = 55	CS *N* = 160	*P*	Emergency CS *N* = 113	Elective CS *N* = 47	*P*
Age (y)	34.1 (3.4)	33.0 (5.3)	0.08	32.8 (5.5)	33.6 (4.8)	0.35
BMI (kg/m^2^)	24.6 (6.7)	24.7 (5.5)	0.55	24.1 (4.7)	25.8 (6.8)	0.37
Nulliparity	13 (24.1%)	72 (45.3%)	**< 0.001**	61 (54.0%)	11 (23.4%)	**< 0.001**
Gestational age (w)	39.4 (2.6)	38.2 (3.4)	**< 0.05**	38.5 (3.7)	37.5 (2.5)	**< 0.001**
Previous CS	18 (34.0%)	52 (32.7%)	0.87	23 (20.4%)	29 (61.7%)	**< 0.001**
Hospital admission (d)	11.7 (13.1)	14.4 (10.9)	**< 0.05**	14.6 (10.5)	13.8 (11.9)	0.18
Blood loss (mL)	5556 (4532)	4262 (3432)	0.053	4166 (3342)	4303 (3486)	0.81
Units of RBC (n)	12.4 (9.4)	10.8 (9.0)	0.19	11.6 (9.6)	9.1 (7.1)	0.18
SAMM before birth (n)	3 (5.5%)	11 (6.9%)	0.52	10 (8.9%)	1 (2.1%)	0.275

*CS* cesarean section, *VD* vaginal birth, *RBC* red blood cells. Data is presented as mean (SD) or number (%)

SAMM occurred before childbirth in 14 (6.5%) and after childbirth in 198 (92.1%) women; in three women this information was unknown (Table 2). In 99 women (46.0%), the indication for laparotomy after birth was intra-abdominal bleeding, followed by severe postpartum hemorrhage (83 women, 38.6%) (Table 3). For cesarean section, the main indication was intra-abdominal bleeding (93 women, 58.1%). For vaginal birth, main indications were severe postpartum hemorrhage (34 women, 61.8%) or suspected uterine rupture (12 women, 21.8%).

**Table 3:** Detailed information of laparotomies after childbirth

Total		VD *N* = 55	CS *N* = 160	*P*	Emergency *N* = 113	Elective *N* = 47	*P*
Indication^*^	Intra-abd. Bleeding	6 (10.9)	93 (58.1)	**< 0.001**	65 (57.5)	28 (59.6)	0.777
	sPPH	34 (61.8)	49 (30.6)		36 (31.9)	13 (27.7)	
	Suspected rupture	12 (21.8)	1 (0.6)		1 (0.9)	0 (0.0)	
	Sepsis	4 (7.2)	7 (4.4)		6 (5.3)	1 (2.1)	
	Hematoma	0 (0.0)	4 (2.5)		3 (2.7)	1 (2.1)	
	Miscellaneous	9 (16.4)	11 (7.5)		6 (5.3)	5 (10.6)	
	Unknown	0 (0.0)	1 (0.6)		1 (0.9)	0 (0.0)	
Time^*^	< 24 h	46 (83.6)	101 (63.1)	**< 0.05**	71 (62.8)	30 (63.8)	**< 0.05**
	2-7d	5 (9.1)	43 (26.9)		30 (26.5)	13 (27.7)	
	>7d	4 (7.3)	12 (7.5)		11 (9.7)	1 (2.1)	
	Unknown	0 (0.0)	4 (2.9)		1 (0.9)	3 (6.4)	
Intervention^*^	Arrest of bleeding:						
	-Abdominal wall	0 (0.0)	13 (8.1)	**< 0.001**	10 (8.9)	3 (6.4)	0.591
	-Intra-abdominal	13 (23.6)	40 (25.0)		28 (24.8)	12 (25.5)	
	-CS scar	2 (3.6)	32 (20.0)		22 (19.5)	10 (21.3)	
	B-lynch procedure	1 (1.8)	8 (5.0)		7 (6.2)	1 (2.1)	
	Ligation	6 (10.9)	11 (6.9)		8 (7.1)	3 (6.4)	
	Hysterectomy	31 (56.4)	32 (20.0)		21 (18.6)	11 (23.4)	
	Drainage	3 (5.5)	9 (5.6)		7 (6.2)	2 (4.3)	
	Negative	2 (3.6)	19 (11.9)		16 (14.2)	3 (6.4)	
	Miscellaneous	10 (18.2)	24 (15.0)		18 (15.9)	6 (12.8)	
	Unknown	0 (0.0)	6 (3.8)		3 (2.7)	3 (6.4)	
Number	1	43 (78.2)	129 (80.6)	0.26	88 (77.9)	41 (87.2)	0.44
	≥2	10 (18.2)	30 (18.8)		24 (21.2)	6 (12.8)	
	Unknown	2 (3.6)	1 (0.6)		1 (0.9)	0 (0.0)	

*CS* cesarean section, *VD* vaginal birth, *sPPH* severe postpartum hemorrhage. Data is presented as number (%)

^*^for 1st laparotomy

A total of 147 (68.4%) laparotomies were performed within 24 h after birth (cesarean section 63.1% vs. vaginal birth 83.6%; *p* < 0.05). Late laparotomies (within 2-7 days) were more likely to happen following cesarean section (26.9% vs. vaginal birth 9.1%; *p* < 0.05).

During the first laparotomy, hysterectomy was the most frequently performed intervention (63 women, 29.3%), followed by control of intra-abdominal (53 women, 24.7%) and caesarean scar-related bleeding (34 women, 15.8%). In 21 (9.8%) women, no therapeutic intervention was done during laparotomy.

Forty out of the 215 women included in the risk analysis (18.6%) had more than one laparotomy: 32 out of these 40 (80.0%) had two, seven (17.5%) had three and one (2.5%) had four laparotomies. In 21 (52.5%) of these 40 women, the operation was due to intra-abdominal bleeding and in 5 (12.5%) re-laparotomy resulted in hysterectomy.

Three out of the 215 women died shortly after or during laparotomy (case fatality rate 1.4%): one woman died in the intensive care unit after hysterectomy for severe hemorrhage following vaginal birth. Another woman, who had a history of cardiac disease, died due to massive intra-peritoneal hemorrhage from iatrogenic perforation of the iliac artery during uterine embolization following vaginal birth. Laparotomy was performed as a last resort, but she died shortly afterwards in the intensive care unit. The third maternal death was due to puerperal sepsis with group-A streptococcus. The woman had delivered a stillbirth vaginally and suffered persistent postpartum hemorrhage despite embolization. She died during hysterectomy.

## Discussion

This study, using a nationwide cohort of women who suffered SAMM, is the first to report national incidence rates of laparotomy after vaginal and cesarean birth. The risk of postpartum laparotomy was more than 16 times higher in women who gave birth by cesarean section compared to those who gave birth vaginally. The risk for laparotomy is lower when cesarean section is planned, but nevertheless still 10 times higher compared to vaginal birth.

Our results also indicate that laparotomy after childbirth may be an appropriate indicator of severe maternal outcome and quality marker for obstetric care. For example, 183 of 215 women (85.1%), fulfill the WHO Maternal Near Miss criterion of having had five or more units of blood transfused [1]. Based on a previously performed hypothetical experiment based study, 113 out of the 215 women (52.6%) would have died if massive blood transfusion had not been available, as is the case in many low-income countries [15].

The rate of laparotomy after cesarean section in women with SAMM in the Netherlands (0.3%) appears relatively low compared to the literature (0.2-0.9%) [2–11]. Since laparotomy after vaginal birth has not been studied before, the incidence we found for laparotomy following vaginal birth cannot be compared to other studies. The largest study of laparotomy following cesarean section was conducted in a single university medical center in Israel and included 80 women over a period of 20 years. Our study is unique because of its large sample size (*n* = 215), included in a relatively short time frame, and its prospective nationwide design.

Postpartum hemorrhage, placental abruption, uterine rupture and previous cesarean section were previously found to be associated with increased risk of re-laparotomy [2, 4, 5, 10]. We confirmed that the main proportion (68.4%) of all laparotomies was performed within 24 h after birth due to either intra-abdominal bleeding (46.0%) or postpartum hemorrhage (38.6%). One third of women (32.6%) had a previous cesarean section. Although placental abruption was not an endpoint, the majority of these cases are likely represented in the group of major obstetric hemorrhage since women would generally receive at least four units of blood. Thirteen women underwent laparotomy due to (suspected) uterine rupture. Infection or sepsis were not reported as outcomes of interest in previous studies. In our study, sepsis was the indication for laparotomy in 11 cases.

Our results need to be interpreted with caution since our study has several important limitations. First, the data from the LEMMoN-study are rather old and changes in incidence and risk may have occurred since data collection took place. However, we are not aware of any currently ongoing studies of postpartum laparotomy and think that our data are therefore still of considerable importance, since more up-to-date information is unlikely to become available for some time. A second limitation is that laparotomy was not a separate inclusion criterion as having severe acute maternal morbidity. This may introduce selection bias, since women who were transfused less than five units of blood, those who did not have hysterectomy, embolization, or uterine rupture and those who were not admitted into intensive care may have been missed. These women would only have been included if the treating obstetrician still decided to include her as severe acute maternal morbidity. Nevertheless, the fact that laparotomies in women with SAMM will have been included validates our conclusions for this group. The fact that the overwhelming majority (149, 93.1%) of SAMM in our cohort occurred after birth provides an additional argument for the hypothesis that SAMM may often be related to the mode of birth. Some of these SAMM conditions may be more common after (difficult) cesarean versus vaginal birth and this is precisely what should be included in any clinical counseling about risks of cesarean section. We analyzed all vaginal births as one group and did not subdivide between instrumental and spontaneous births, postulating that the risk of laparotomy following a successful instrumental birth would not be elevated.

With regard to mode of birth (vaginal birth, emergency and planned cesarean section) there are some noteworthy results. In contrast with what is commonly assumed, the proportion of re-laparotomy due to intra-abdominal bleeding was comparable for planned and emergency cesarean section. The timing to perform laparotomy is more often between two and seven days after cesarean section than after vaginal birth, where laparotomy is performed earlier. In total, 140 out of 160 (88%) laparotomies after cesarean birth were performed within four days. This means that clinicians should be particularly cautious of the occurrence of complications that may lead to laparotomy in the first four days after cesarean section. It should also be underlined that almost 20% of women had more than one laparotomy after birth and that in 10% of all laparotomies exploration was performed without any therapeutic intervention.

Cesarean birth rates have been increasing for the past decades up to 47.6% in China and 50% in Brazil [16, 17]. In the Netherlands, although rates are relatively low, the proportion of cesarean section has risen from 11% to 16% between 1999 and 2012 [18]. A recent study in China showed that 40% of cesarean sections were performed without medical indication [19]. Considering the elevated risk of laparotomy after cesarean section, such developments will inevitably lead to a rise in unfavorable outcomes. This adds to the results of previous studies in which cesarean birth was also found to be associated with a clearly elevated risk of maternal morbidity and mortality compared to vaginal birth, regardless of the indication [12, 13, 20]. Our study addresses both short- and long-term adverse effects of cesarean section: the complications as a result of initial surgery requiring laparotomy, and the complications in subsequent pregnancies, such as abnormally invasive placentation and the risks of birth in presence of a uterine scar [21–23]. Women with vaginal birth after previous cesarean section are over-represented (18/55 women, 34.0%) compared to the general Dutch pregnant population (6.0%) [14].

WHO has recently stated again that national cesarean birth rates above 10% are not associated with a further decrease in maternal or neonatal mortality [24]. It is alarming that cesarean rates are still on the rise in most countries [16]. These rates may be difficult to curb, but it is important to realize that every cut may have its cost. Adverse maternal outcome, including laparotomy, should be kept in mind when cesarean section is considered and women are counselled for mode of birth, particularly when maternal request is the only indication.

## Conclusion

Main finding of this nationwide cohort study is that the risk of postpartum laparotomy in women with severe acute maternal morbidity in the Netherlands was much higher after cesarean section compared to vaginal birth. This information must be taken into account by clinicians when considering mode of birth and can be interpreted as yet another reason to reduce unnecessary cesarean sections.

## Abbreviations

CI: Confidence interval
RR: Relative risk
SAMM: Severe acute maternal morbidity
WHO: World Health Organization

## References

[1] World Health Organization, Department of Reproductive Health and Research (2011). Evaluating the quality of care for severe pregnancy complications. The WHO near-miss approach for maternal health.

[2] Ashwal E, Yogev Y, Melamed N, Khadega R, Ben-Haroush A, Wiznitzer A, Peled Y (2014). Characterizing the need for re-laparotomy during puerperium after cesarean section. Arch Gynecol Obstet.

[3] Gedikbasi A, Akyol A, Asar E, Bingol B, Uncu R, Sargin A, Ceylan Y (2008). Re-laparotomy after cesarean section: operative complications in surgical birth. Arch Gynecol Obstet.

[4] Kessous R, Danor D, Weintraub YA, Wiznitzer A, Sergienko R, Ohel I, Sheiner E (2012). Risk factors for relaparotomy after cesarean section. J Matern Fetal Neonatal Med.

[5] Levin I, Rapaport AS, Salzer L, Maslovitz S, Lessing JB, Almog B (2012). Risk factors for relaparotomy after cesarean birth. Int J Gynaecol Obstet.

[6] Lurie S, Sadan O, Golan A (2007). Re-laparotomy after cesarean section. Eur J Obstet Gynecol Reprod Biol.

[7] Ragab A, Mousbah Y, Barakat R, Zayed A, Badawy A (2014). Re-laparotomy after cesarean births: risk factors and how to avoid?. J Obstet Gynaecol.

[8] Seal SL, Kamilya G, Bhattacharyya SK, Mukherji J, Bhattacharyya AR (2007). Relaparotomy after cesarean birth: experience from an Indian teaching hospital. J Obstet Gynaecol Res.

[9] Seffah JD (2005). Re-laparotomy after cesarean section. Int J Gynaecol Obstet.

[10] Shinar S, Hareuveni M, Ben-Tal O, Many A (2013). Relaparotomies after cesarean sections: risk factors, indications, and management. J Perinat Med.

[11] Levitt L, Sapir H, Kabiri D, Ein-Mor E, Hochner-Celnikier D, Amsalem H (2015). Re-laparotomy following cesarean birth - risk factors and outcomes. J Maternal Fetal Neonatal Med.

[12] Hall MH, Bewley S (1999). Maternal mortality and mode of birth. Lancet.

[13] van Dillen J, Zwart JJ, Schutte J, Bloemenkamp KW, van Roosmalen J (2010). Severe acute maternal morbidity and mode of birth in the Netherlands. Acta Obstet Gynecol Scand.

[14] Zwart JJ, Richters JM, Ory F, de Vries JI, Bloemenkamp KW, van Roosmalen J (2008). Severe maternal morbidity during pregnancy, birth and puerperium in the Netherlands: a nationwide population-based study of 371,000 pregnancies. BJOG.

[15] Hendriks J, Zwart JJ, Briët E, Brand A, van Roosmalen J (2013). The clinical benefit of blood transfusion: a hypothetical experiment based on a nationwide survey of severe maternal morbidity. Vox Sang.

[16] Vogel JP, Betrán AP, Vindevoghel N, Souza JP, Torloni MR, Zhang J, Tunçalp Ö, Mori R, Morisaki N, Ortiz-Panozo E, Hernandez B, Pérez-Cuevas R, Qureshi Z, Gülmezoglu AM (2015). Temmerman M; WHO multi-country survey on maternal and newborn Health Research network. Use of the Robson classification to assess caesarean section trends in 21 countries: a secondary analysis of two WHO multicountry surveys. Lancet Glob Health.

[17] Ramires de Jesus G, Ramires de Jesus N, Peixoto-Filho F, Lobato G (2015). Cesarean rates in Brazil: what is involved?. BJOG.

[18] Stichting Perinatale Registratie Nederland. Perinatale Registratie Nederland Grote Lijnen 1999 - 2012 [The Netherlands Perinatal Registry Trends 1999-2012]. Utrecht: Stichting Perinatale Registratie Nederland; 2011:26-27.

[19] Deng W, Klemetti R, Long Q, Wu Z, Duan C, Zhang WH, Ronsmans C, Zhang Y, Hemminki E (2014). Cesarean section in shanghai: women's or healthcare provider's preferences?. BMC Pregnancy Childbirth.

[20] Schuitemaker N, van Roosmalen J, Dekker G, van Dongen P, van Geijn H, Gravenhorst JB (1997). Maternal mortality after cesarean section in The Netherlands. Acta Obstet Gynecol Scand.

[21] Nederlandse Vereniging voor Obstetrie & Gynecologie. Zwangerschap en bevalling na een voorgaande sectio cesarea [Pregnancy and birth after previous cesarean section]. [http://www.nvog-documenten.nl/]. Accesed 30 Jul 2016.

[22] van Ham MA, van Dongen PW, Mulder J (1997). Maternal consequences of cesarean section. A retrospective study of intra-operative and postoperative maternal complications of cesarean section during a 10-year period. Eur J Obstet Gynecol Reprod Biol.

[23] Zelop C, Heffner LJ (2004). The downside of cesarean birth: short- and long-term complications. Clin Obstet Gynecol.

[24] Ye J, Zhang J, Mikolajczyk R, Torloni MR, Gulmezoglu AM, Betran AP (2015). Association between rates of cesarean section and maternal and neonatal mortality in the 21st century: a worldwide population-based ecological study with longitudinal data. BJOG.

